# Application of stool-PCR for the diagnosis of *Helicobacter pylori* from stool in Nigeria- a pilot study

**DOI:** 10.1186/2193-1801-1-78

**Published:** 2012-12-22

**Authors:** Stella I Smith, Muinah A Fowora, Olufunmilayo A Lesi, Elizabeth Agbebaku, Peter Odeigah, Fatimah B Abdulkareem, Charles A Onyekwere, Chimere A Agomo, Monica Contreras

**Affiliations:** 1 Molecular Biology and Biotechnology Division, Nigerian Institute of Medical Research, 6 Edmond Crescent, Yaba, PMB 2013, Lagos, Nigeria; 2 Department of Medicine, College of Medicine, University of Lagos, Lagos, Nigeria; 3 Department of Cell Biology and Genetics, University of Lagos, Lagos, Nigeria; 4 Department of Pathology, College of Medicine, University of Lagos, Lagos, Nigeria; 5 Department of Medicine, Lagos State University Teaching Hospital (LASUTH), Ikeja, Nigeria; 6 Biochemistry and Nutrition Division, Nigerian Institute of Medical Research, 6 Edmond Crescent, Yaba, Lagos, Nigeria; 7 Instituto Venezolano de Investigaciones Científicas, Caracas, Venezuela

**Keywords:** UBT, Stool-PCR, *Helicobacter pylori*, Nigeria

## Abstract

There are various methods for detection of *Helicobacter pylori* and the gold standard for non-invasive detection is the urea breath test (UBT). The aim of the study is therefore to detect *H. pylori* from the stool of patients with dyspepsia by PCR and compare results obtained with UBT. A total of 97 stool samples from patients presenting with dyspeptic symptoms in Lagos University Teaching Hospital (LUTH) were screened for urea breath test (UBT) and the presence of *H. pylori* DNA using stool-PCR. Out of 97 stool samples analysed, 38 (39.2%) were positive for *Helicobacter* spp. and 20 (20.6%) positive for *H. pylori* by PCR, through amplification of 16S rRNA and *glmM*genes respectively. Of the 20 positive by *glm*M gene, the *cagA*gene was detected in 8 (40%) samples, while 47 (48.5%) out of 97 stool samples were positive for *H. pylori* by UBT. The sensitivity and specificity of the *glmM* gene compared with UBT as the gold standard is 42.6% and 100% respectively. The positive predictive value (PPV) was 100% while the negative predictive value (NPV) was 60%.The method may be useful for detecting *H. pylori* from stool amongst children especially where most hospitals lack endoscope for children although the method is expensive.

## Introduction

*Helicobacter pylori* is the causative agent of gastritis, peptic ulcer disease (PUD), MALT lymphoma and a risk factor in the development of gastric cancer (Blaser and Berg 2001). It has also been classified as a class I carcinogen by the International Agency for Research on Cancer (IARC) Monogr Eval Carcinog Risks Hum 1994(an arm of WHO) in 1994. The mode of transmission still unknown, although epidemiologic studies suggest close person to person contact, intrafamilial spread. There is still uncertainty whether transmission occurs primarily through faecal-oral or gastric-oral route (Covacci *et al*., 1999). There are various tests for *H. pylori* diagnoses broadly categorized into two: invasive and non-invasive (Mégraud and Lehours^2007^). Invasive methods require endoscopy while the non-invasive methods do not. The invasive methods include culture (which is the gold standard), Rapid urease tests (RUT), histology, direct gram stain, PCR based methods and fluorescence *in situ* hybridization (FISH). The non-invasive methods include serology (which does not measure active infection), urea breath tests (UBT) (both ^13^C and ^14^C, gold standard, which is an expensive test and cannot be available in routine clinical laboratories), *Helicobacter pylori* stool antigen tests (HpSA) (Mégraud and Lehours 2007).

*Helicobacter pylori* can be detected in stool specimens either by culture (difficult due to diverse microorganisms in the stool and fastidious nature of *H. pylori*), HpSA (discrepancies also occur from one geographical area to the other) and stool-PCR (with success rates of 25%–100%). Generally, the differences in detection rate of *H. pylori* in stool is due to *H. pylori* degradation in the gastrointestinal tract and/or presence of inhibitors such as complex polysaccharides and also its presence in low concentration in stools (Kabir 2001).

Many PCR methods have been developed to detect the organism directly in different clinical specimens. Various authors have reported on the use of stool-PCR for diagnosis of *H. pylori* (Şen et al. 2005; Hirai et al. 2009; Aktepe et al. 2011). The sensitivity with this method in recent times has varied from 21%–65.22% (Aktepe et al.^2011^; Şen et al. 2005). However non-invasive methods that give accurate results are sought after. The study is therefore aimed at detecting *H. pylori* in stools of patients with dyspepsia using stool-PCR.

## Materials and methods

Study period: August 2009–April 2010.

Ethical approval was obtained from NIMR-IRB, while informed consent was obtained from patients before stool samples were taken.

Inclusion Criteria: patients not on antibiotics or PPI’s/histamine- 2- receptor blockers at least a month before the study as well as presenting with dyspepsia symptoms.

Exclusion Criteria: those currently on antibiotics and or PPI’s/histamine 2 receptor blockers.

### Clinical samples

Sampling method: Convenient sampling. Stool samples from 97 dyspeptic patients at the LUTH were used for this study. The stool samples were collected using sterile toothpicks into eppendorf tubes containing 700 μl absolute ethanol at room temperature. The samples was considered *H*. *pylori*- positive when *glmM* gene or both genes (*glmM* and *cagA*) were detected by PCR, and the results compared with those from UBT as the gold standard and confirmatory test.

### UBT

To screen for UBT, *H*. *pylori* testing was performed using a validated Heliprobe system (Noster AB Sweden). The heliprobe UBT is a ^14^C-based urea breath test for the detection of *H. pylori* infection. It comprises the Helicap TM capsule which contains the ^14^C-labelled urea which is swallowed and metabolized to carbon dioxide and ammonia by the urease enzyme produced by *H. pylori*. Labeled ^14^ C exhaled in the breath is captured by a breathcard and analysed by the automatic heliprobe analyser. Levels above 50 counts/min were considered positive of *H. pylori* infection.

### Stool-PCR

#### 16SrRNA gene (399 bp)

Briefly, DNA from the stool samples were purified using the QIAamp® DNA Stool Mini kit (Hilden, Germany) containing an inhibitEX (removes all DNA damaging substances and PCR inhibitors in stool), and detected using a PCR assay targeting a 399 bp fragment of the 16S rRNA gene of *Helicobacter* spp. with two specific primers, *Heli*F (AAC GAT GAA GCT TCT AGC TTG CTA G) and *Heli*R (GTG CTT ATT CST NAG ATA CCG TCA T) (Germani et al. 1997). PCR was performed using the Ready To-Go PCR beads kit by GE Healthcare (Buckinghamshire, UK). Amplification was carried out in an Eppendorf Mastercycler gradient (Hamburg, Germany) using the following cycling parameters: An initial denaturation at 94°C for 5 min and 35 cycles of 94°C for 30 s, 56°C for 1 min and 72°C for 1 min. This was followed by a final extension of 72°C for 10 min.

The PCR product was separated on a 2% Agarose gel and 50 bp ladder was used as DNA molecular weight standard.

#### *cag*A gene (128 bp)

PCR amplification using this primer was carried out using the primer set cagA-F : 5^′^-ATAATGCTAAATTAGACAACTTGAGCGA-3^′^ and 5^′^-AGAAACAAAAGCAATACGATCATTC-3^′^ Rugge et al. (1999). The 25 μl reaction mixture consisted of x1 PCR buffer, 1.5 mM Magnesium Chloride, 200 μM of each dNTP, 20pmol of each primer and 1U Taq DNA polymerase (Promega).

Amplification was carried out in an Eppendorf Mastercycler gradient using the following cycling parameters: an initial denaturation at 94°C for 5 min and 40 cycles of 94°C for 1 min, 54°C for 1 min and 72°C for 1 min. This was followed by a final extension of 72°C for 5 min. The PCR product was separated on a 2% Agarose gel and 50 bp ladder was used as DNA molecular weight standard.

#### *glmM* gene (294 bp)

The following primers were used: F : 5^′^-GGATAAGCTTTTAGGGGTGTTAGGGG-3^′^ (738–763) and R : 5^′^-GCTTACTTTCTAACACTAACGCGC-3^′^(1010–1033) Kansau et al. (1996). The 25 μl reaction mixture consisted of x1 PCR buffer, 1.5 mM Magnesium Chloride, 200 μM of each dNTP, 20pmol of each primer and 1U Taq DNA polymerase (Promega).

The following were the conditions for amplification: one cycle of denaturation at 94°C × 5 min; 35 cycles at 94°C × 1 min, annealing at 56°C × 1 min, and elongation at 72°C × 2 min, followed by a final elongation step by 1 cycle at 72°C × 7 min. 15°C and amplification was carried out in an Eppendorf Master cycler gradient (Hamburg). The PCR product was separated on a 2% Agarose gel and 50 bp ladder was used as DNA molecular weight standard.

Sensitivity of the primers was determined by testing other bacterial strains from related genus e.g. *Salmonella* Typhimurium and *E*. *coli*. The results of the *glm*M gene was compared with the gold standard (UBT) using the positive and negative predictive values and also the sensitivity and specificity.

The *glm*M and *cag*A genes are genes commonly used for the diagnosis of *H. pylori* directly from biopsy specimens as well as isolates and was therefore adapted for use for stool PCR in this study.

## Results

None of the *Salmonella Typhimurium* or *E. coli* amplified with the set of specific primers for 16S rRNA and *glmM* genes.

Out of 97 stool samples analysed, *Helicobacter* spp. and *H. pylori* DNA were detected in 38 (39.2%) and 20 (20.6%) by 16S rRNA and *glm*M genes respectively. The detection of *cagA* positivity by PCR was observed in 8 out of 20 (40%) samples positive for *glm*M gene whereas 47 out of 97 (48.5%) samples were positive for *H. pylori* by UBT (Table 1). Those simultaneously positive for stool-PCR (*glmM* gene) and UBT were 20 (42.6%) while 27 (57.5%) were positive for UBT and negative for stool-PCR using *glmM* gene. Table 2 shows the evidenced genes (*cag*A and *glm*M) and the subspecies. The sensitivity and specificity of the *glmM* gene compared with UBT as the gold standard is 42.6% and 100% respectively. The positive predictive value (PPV) was 100% while the negative predictive value (NPV) was 60%.

**Table 1:** **Results of stool PCR test using*glmM*gene with UBT as the gold standard**

		Results for stool-PCR, ***glm***M gene (n = 97)						
		Positive	Negative	Total	Sensitivity	Specificity	PPV	NPV
UBT (n = 97)	Positive	20	27	**47**	42.60%	100%	100.00%	60.00%
	Negative	0	50	**50**				
	Total	**20**	**77**	**97**

**Table 2:** **shows*cag*A +*glm*M genes with the subspecies,*Helicobacter*spp. and*H. pylori***

	***Helicobacter*** spp. (16S rRNA)	***H. pylori***
*cag*A+*glm*M	8	8
*glm*M	10	12
*cag*A, *glm*M -	20	-
Total	38	20

**Figure 1:**
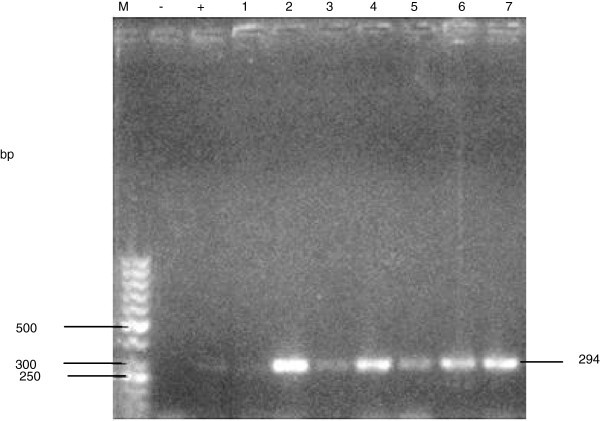
**Gel electrophoresis of PCR products amplified using the glmM gene for H. pylori. Lanes: M: molecular weight marker; -: negative control, +: positive control; 1 – 7 samples.**

**Figure 2:**
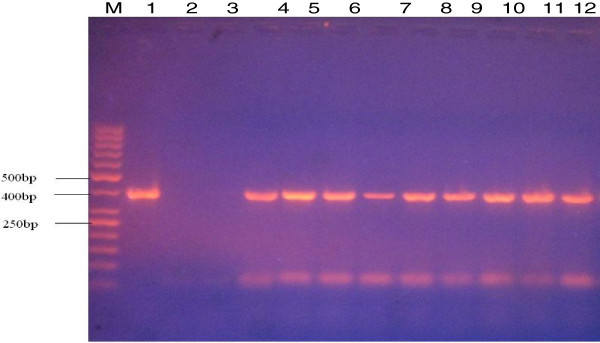
**Gel electrophoresis of PCR products amplified using the 16S rRNA gene of Helicobacter spp. Lanes**: M: molecular weight marker; 1: positive control, 2: negative control; 3 – 12 samples.

Figure 1 and 2 shows the PCR amplification of the samples using *glm*M and 16S rRNA genes respectively.

## Discussion

Non-invasive tests can play an important role in the diagnosis of *H. pylori* infections. However, all have their limitations in terms of cost, specificity, and sensitivity. The data presented here, are the results of a pilot study that preceded a project encompassing for UBT and PCR in monitoring a larger dyspeptic patient population. The 16S rRNA PCR amplified *Helicobacter* DNA (39%) in stool samples, being the most widely used method for detection of *H. pylori* in clinical specimens. However, the *glmM* gene PCR is the most sensitive and specific for the detection of *H. pylori* in gastric biopsy specimens Lu et al. (1999). The presence of *H. pylori* DNA was found in stool samples (21%). The results show that to some extent the *glmM* gene for detection of *H*. *pylori* DNA by PCR may be useful for the diagnosis of *H. pylori* from stool. In comparison with UBT as the gold standard, the sensitivity and specificity was 42.6% and 100%, respectively. The sensitivity is low. In a related study by Aktepe et al. (2011); using five methods of detection of *H. pylori*, the stool-PCR had the lowest sensitivity (21%). However, Makristathis et al. (1998) developed a semi-nested PCR assay, targeted to a species-specific protein antigen which is present in all strains of *H. pylori*, for detection and follow-up of *H. pylori* infected patients and the sensitivity was as high as 93.7% and the specificity was 100%. Another study by Mishra et al. (2008) corroborated the high sensitivity of stool-PCR in both pre-eradication and post-eradication to be 72.5% and 97.1% and the authors concluded that stool-PCR was better indicator than HpSA test in post-eradication assessment of infection. Another report by Gramley et al. (1999) reported sensitivity of stool-PCR to be 73%.

In this study, *cag*A gene was present in 40% of the samples positive for *H. pylori* by *glm*M gene and in a related study by Bindayna et al. (2006), *cag*A gene was found to be present in 22.7% this is lower than in our study. *cag*A positive *H. pylori* strains have been associated with severity of disease outcome and plays a critical role in the development of stomach cancer. From our previous studies with gastric biopsy specimens, *cag*A positive *H. pylori* was found in 68% of the biopsy specimens (Smith *et al*. 2004); while from a related study by Bindayna et al. (2006) 59% of the biopsy samples were positive for *cag*A gene, when compared with 22.7% from stool samples. It goes to show the low prevalence of *cag*A gene in stool samples when compared to biopsy samples and this could be attributable to low numbers of *H. pylori* in stool samples as well as problems of faecal PCR inhibitors although the Qiagen kit was used to circumvent this in our study. In terms of high sensitivity and specificity found in studies with stool PCR these could be due to methods of DNA extraction as well as different targets used for detection of *H. pylori* by PCR. In addition, *glmM* and *cagA* genes have not been found yet in *Helicobacter* spp. other than *H. pylori*, suggesting that non-*H. pylori* helicobacters species may be commonly found in the stool of humans.

The PCR using *H. pylori* species specific primers in the long run could be also useful especially when infants, or very young children and patients with certain neurological disorders are being screened and UBT cannot be convenient for them. The method is also convenient for sample collection and samples can even be collected at home.

The method using the Qiagen stool mini kit is expensive but inexpensive methods that could help remove inhibitors in stool could be used to reduce cost e.g. filteration of stool and column chromatography.

From our study this method is not suitable for routine clinical setting in the developing countries but could be restricted to Reference labs where issues bordering on recurrent *H*. *pylori* infections and treatment failure are a problem especially where culture is difficult due to power outages in the developing countries. This study did not look at very young children due to the convenient sampling method which was biased.

The advantages and disadvantages of this method in our lab is as follows:

Advantages of stool-PCR

1. It is non-invasive
2. It can be used in children and most especially those with neurological disorders.
3. It is useful for early screening in children to enable prompt detection of *H. pylori* related infection and aid in subsequent treatment.
4. It would capture the generality of children, especially the under 10, as most hospitals don’t have child endoscopes so it becomes difficult to screen children thoroughly due to lack of child endoscopes.

Disadvantages

1. It is expensive for use in developing countries as long as the qiagen kit is employed.
2. It might not be readily available in most diagnostic labs as with other PCR related methods and so has to be restricted to reference labs.

In conclusion, it could be most useful for diagnosis of *H*. *pylori* in children especially as endoscope for children is lacking.

## References

[CR1_59] Aktepe OC Ciftci IH Safak B Uslan I Dilek FH Five methods for the detection of Helicobacter pylori in the Turkish population World J Gastroenterol 2011 17 5172 5176 10.3748/wjg.v17.i47.5172 22215941 PMC3243883

[CR2_59] Bindayna KM Al Baker WA Botta GA Detection of Helicobacter pylori cagA gene in gastric biopsies, clinical isolates and faeces Indian J Med Microbiol 2006 24 195 200 16912439

[CR3_59] Blaser MJ Berg DE Helicobacter pylori genetic diversity and risk of human disease J Clin Invest 2001 107 767 773 10.1172/JCI12672 11285290 PMC199587

[CR4_59] Covacci A Telford JL Del Giudice G Parsonnet J Rappuoli R Helicobacter pylori virulence and genetic geography Science 1999 284 1328 1333 10.1126/science.284.5418.1328 10334982

[CR5_59] Germani Y Dauga C Duval P Strategy for the detection of Helicobacter species by amplification of 16S rRNA genes and identification of H. felis in a human gastric biopsy Res Microbiol 1997 148 315 326 10.1016/S0923-2508(97)81587-2 9765810

[CR6_59] Gramley WA Ashgar A Frierson HF Powell SM Detection of Helicobacter pylori DNA in faecal samples from infected individuals J Clin Microbiol 1999 37 2236 2240 10364591 10.1128/jcm.37.7.2236-2240.1999 PMC85126

[CR7_59] Hirai I Sasaki T Kimoto A Fujimoto S Moriyama T Yamamoto Y Assessment of East-Asian type cagA positive Helicobacter pylori using stool specimens from asymptomatic healthy Japanese individuals J Med Microbiol 2009 58 1149 1153 10.1099/jmm.0.010934-0 19528144

[CR8_59] Schistosomes, liver flukes and Helicobacter pylori 1994 Lyon IARC Working Group on the Evaluation of Carcinogenic Risks to Humans PMC7681621 7715068

[CR9_59] Kabir S Detection of Helicobacter pylori in faeces by culture, PCR and enzyme immunoassay J Med Microbiol 2001 50 1021 1029 11761185 10.1099/0022-1317-50-12-1021

[CR10_59] Kansau I Raymond J Bingen E Genotyping of Helicobacter pylori isolates by sequencing of PCR products and comparison with the RAPD technique Res Microbiol 1996 147 661 669 10.1016/0923-2508(96)84023-X 9157493

[CR11_59] Lu JJ Perng CL Shyu RY Comparison of five PCR methods for detection of Helicobacter pylori DNA in gastric tissues J Clin Microbiol 1999 37 772 774 9986850 10.1128/jcm.37.3.772-774.1999 PMC84550

[CR12_59] Makristathis A Pasching E Schutze K Wimmer M Rotter ML Hirschl AM Detection of Helicobacter pylori in stool specimens by PCR and antigen enzyme immunoassay J Clin Microbiol 1998 36 2772 2774 9705436 10.1128/jcm.36.9.2772-2774.1998 PMC105206

[CR13_59] Mégraud F Lehours P Helicobacter pylori detection and antimicrobial susceptibility testing Clin Microbiol Rev 2007 20 280 322 10.1128/CMR.00033-06 17428887 PMC1865594

[CR14_59] Mishra S Singh V Rao G Detection of Helicobacter pylori in stool specimens: comparative evaluation of nested PCR and antigen detection Infect Genet Evol 2008 8 815 819 10.1016/j.meegid.2008.08.001 19738352

[CR15_59] Rugge M Busatto G Cassaro M Patients younger than 40 years with gastric carcinoma: Helicobacter pylori genotype and associated gastritis phenotype Cancer 1999 85 2506 2511 10.1002/(SICI)1097-0142(19990615)85:12<2506::AID-CNCR3>3.0.CO;2-I 10375095

[CR16_59] Şen N Yilmaz Ö Şımşek İ Küpelıoğlu AA Ellıdokuz H Detection of Helicobacter pylori DNA by a Simple Stool PCR Method in Adult Dyspeptic Patients Helicobacter 2005 10 353 359 10.1111/j.1523-5378.2005.00326.x 16104952

[CR17_59] Smith SI Oyedeji KS Arigbabu AO Comparison of three PCR methods and cagA for the detection of H. pylori DNA from gastric biopsies World J Gastroenterol 2004 10 1958 1960 15222045 10.3748/wjg.v10.i13.1958 PMC4572239

